# Vaccination with murid herpesvirus-4 glycoprotein B reduces viral lytic replication but does not induce detectable virion neutralization

**DOI:** 10.1099/vir.0.023085-0

**Published:** 2010-10

**Authors:** Janet S. May, Philip G. Stevenson

**Affiliations:** Division of Virology, Department of Pathology, University of Cambridge, UK

## Abstract

Herpesviruses characteristically disseminate from immune hosts. Therefore in the context of natural infection, antibody neutralizes them poorly. Murid herpesvirus-4 (MuHV-4) provides a tractable model with which to understand gammaherpesvirus neutralization. MuHV-4 virions blocked for cell binding by immune sera remain infectious for IgG-Fc receptor^+^ myeloid cells, so broadly neutralizing antibodies must target the virion fusion complex – glycoprotein B (gB) or gH/gL. While gB-specific neutralizing antibodies are rare, its domains I+II (gB-N) contain at least one potent neutralization epitope. Here, we tested whether immunization with recombinant gB presenting this epitope could induce neutralizing antibodies in naive mice and protect them against MuHV-4 challenge. Immunizing with the full-length gB extracellular domain induced a strong gB-specific antibody response and reduced MuHV-4 lytic replication but did not induce detectable neutralization. gB-N alone, which more selectively displayed pre-fusion epitopes including neutralization epitopes, also failed to induce neutralizing responses, and while viral lytic replication was again reduced this depended completely on IgG Fc receptors. gB and gB-N also boosted neutralizing responses in only a minority of carrier mice. Therefore, it appears that neutralizing epitopes on gB are intrinsically difficult for the immune response to target.

## INTRODUCTION

Herpesviruses are widespread pathogens that use immune evasion to establish persistent infectivity in immunocompetent hosts (Yewdell & Hill, 2002). Most can be neutralized *in vitro*. However, herpesvirus carriers not only remain infectious, but can be superinfected (Gorman *et al.*, 2006) and show little tendency to select viral antigenic variants. Therefore in the context of natural infection, virus neutralization works poorly. Understanding why is important, as this could potentially reveal new means of infection control.

Studies of gammaherpesvirus neutralization have concentrated on Epstein–Barr virus (EBV). Antibodies to gp350 can stop EBV infecting B cells (Thorley-Lawson & Poodry, 1982). However, the gp350-specific antibodies of EBV carriers do not render them non-infectious, and vaccination with gp350 failed to reduce the incidence of EBV infection (Sokal *et al.*, 2007). This could reflect gp350-independent host entry (Janz *et al.*, 2000), which gp350-specific antibodies might even enhance (Turk *et al.*, 2006). Neutralization studies of both EBV and the Kaposi's sarcoma-associated herpesvirus (KSHV) have been hampered by the difficulty of infecting cells at more than a very low level. Thus, an anti-gH/gL mAb can block EBV infection of epithelial cells, an anti-gp42 mAb can block EBV infection of B cells (Li *et al.*, 1995), and KSHV can be neutralized by sera raised against the whole virus (Dialyna *et al.*, 2004), gH/gL (Naranatt *et al.*, 2002), gB (Akula *et al.*, 2001) or K8.1 (Sakamoto *et al.*, 2010), but much of this neutralization has been weak (<threefold reduction in infection) in settings where infection may be suboptimal – for example, EBV infects epithelial cells poorly *in vitro*. Its quantitative significance is therefore hard to assess.

In contrast to EBV and KSHV, murid herpesvirus-4 (MuHV-4) efficiently and productively infects a wide range of cell types (although *in vitro* B-cell infection remains problematic). It also infects mice. However, while antibody reduces MuHV-4 lytic replication *in vivo* (Stevenson *et al.*, 1999a; Kim *et al.*, 2002; Gangappa *et al.*, 2002), such protection depends on IgG Fc receptor engagement rather than neutralization (Wright *et al.*, 2009), perhaps because cell association allows neutralization-resistant virus spread (Stevenson *et al.*, 2009). The likely key setting for MuHV-4 neutralization and its evasion – the transmission of cell-free virions between hosts – is not yet experimentally accessible. Nevertheless, MuHV-4 allows us to explore how gammaherpesvirus neutralization might be improved through immunization.

MuHV-4 infects fibroblasts and epithelial cells by first attaching to heparan sulfate via gp70 (Gillet *et al.*, 2007a) or gH/gL (Gillet *et al.*, 2008a). These interactions can be blocked by mAbs derived from virus carriers, and a block to cell binding is the main mechanism by which immune sera stop MuHV-4 infecting fibroblasts (Gill *et al.*, 2006). However, the apparently neutralized virions can still infect myeloid cells via IgG Fc receptors (Rosa *et al.*, 2007), and the limited capacity of immune sera to block host entry (Gillet *et al.*, 2007b) suggests that an equivalent rescue operates *in vivo*. In contrast to this redundancy in cell binding, infection always requires membrane fusion. Therefore, the virion fusion machinery – gB and gH – is potentially a more universal neutralization target, and mAbs specific for gB or gH/gL (the predominant form of gH on extracellular virions) can block the infection of both fibroblasts and macrophages (Gillet *et al.*, 2007b). However, several factors limit the vulnerability of gB and gH to neutralization.

First, gB and gH/gL are poorly immunogenic in the context of whole virus (Gillet *et al.*, 2007c). Neither is conformationally stable without the other (Gillet *et al.*, 2008b), so native virion epitopes tend to be lost unless the gH/gL/gB complex remains intact. In contrast, native gp150 epitopes are preserved even in small protein fragments (Gillet *et al.*, 2007c). Thus gp150, which is not a neutralization target, dominates the antibody response of MuHV-4 carrier mice. gp150-specific antibodies both drive IgG Fc receptor-dependent infection and tend to suppress neutralizing responses (Gillet *et al.*, 2007c), presumably by competition for antigen. Boosting carrier mice with recombinant gH/gL can overcome the immunodominance of gp150 and improve neutralization titres; boosting with gB works less well (Gillet *et al.*, 2007b).

Second, a complicated entry mechanism allows key fusion complex epitopes to remain hidden on extracellular virions. MuHV-4 infects by endocytosis (Gill *et al.*, 2006). Fusion follows gH/gL dissociation in late endosomes (Gillet *et al.*, 2008b), when gH changes to an antigenically very different form (gH-only). Since gH-only is inaccessible pre-endocytosis, gH-specific antibodies must neutralize by blocking the gH/gL to gH-only transition. gB, which forms a complex with gH/gL (Gillet & Stevenson, 2007a), may also not be fully revealed until after endocytosis (Gillet *et al.*, 2009a). The gB N terminus (gB-NT, residues 25–65) is accessible and is a neutralization target for several herpesviruses (Ohlin *et al.* 1993; Okazaki *et al.*, 2006) including MuHV-4 (Gillet *et al.*, 2006), but is readily masked by *O*-linked glycans, making this neutralization strongly cell type-dependent (Gillet & Stevenson, 2007b).

Most MuHV-4 gB-specific mAbs recognize it either before or after virion endocytosis, consistent with an extensive fusion-associated conformation switch (Gillet *et al.*, 2008c). The recombinant gB extracellular domain (residues 1–724) displays mainly post-fusion epitopes. All known pre-fusion epitopes, including all known neutralization epitopes, lie within residues 1–423 (gB-N) (Gillet *et al.*, 2008c), of which residues 81–423 form gB domains I+II (Heldwein *et al.*, 2006). Most gB-specific neutralizing mAbs have been IgMs and therefore difficult to reproduce by vaccination (Gillet *et al.*, 2006, 2008c). However, we have recently identified a potent neutralizing IgG mAb, SC-9E8, that recognizes gB residues 81–423 (Gillet *et al.*, 2009a). Delivering its epitope could therefore improve MuHV-4 neutralization. We tested here whether recombinant forms of gB could elicit MuHV-4-neutralizing antibodies and protect against MuHV-4 challenge.

## RESULTS

### Protection against primary MuHV-4 lytic replication by vaccinia virus-expressed gB (VAC-gB)

**Fig. 1.**
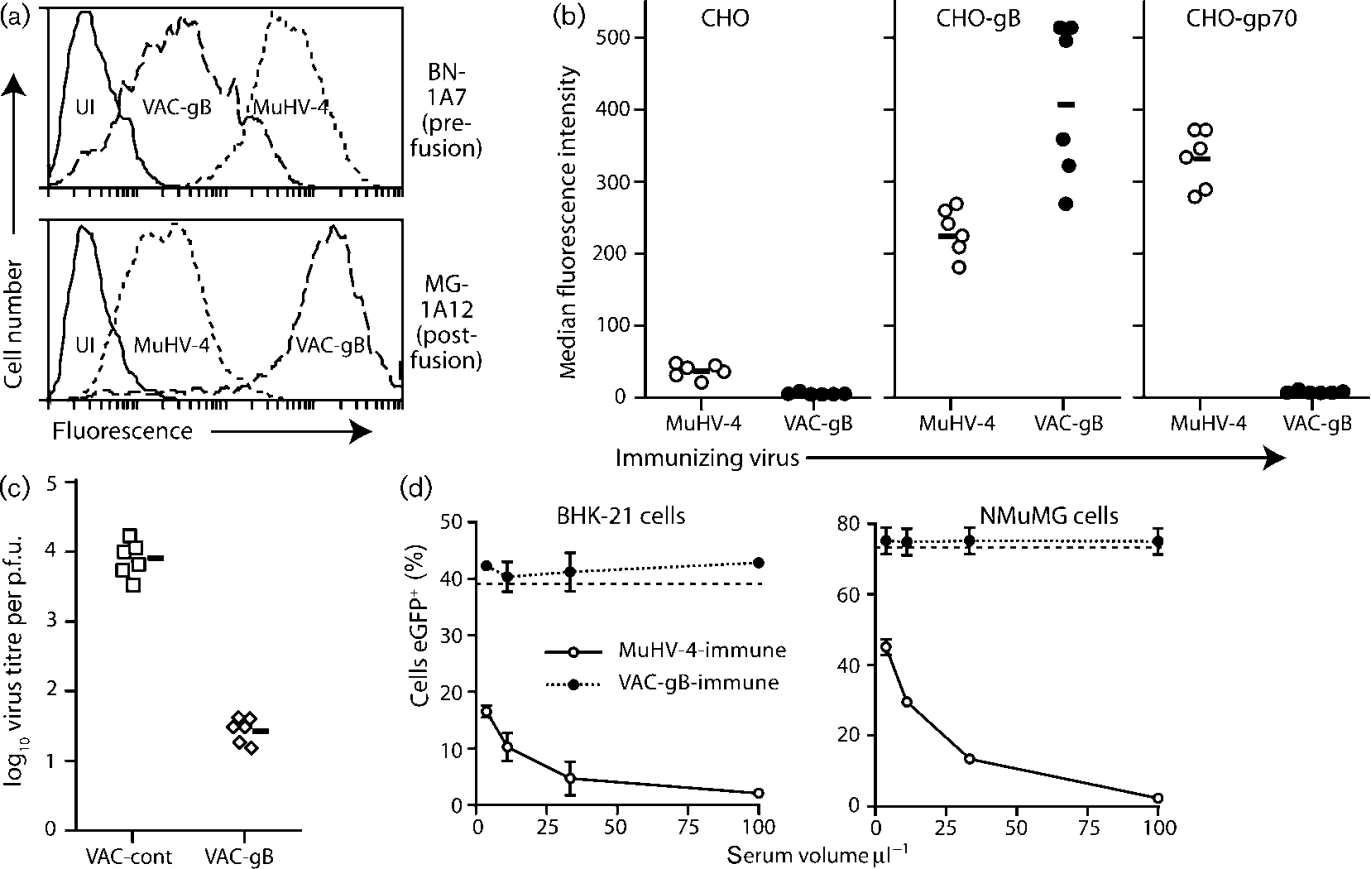
Expression of gB epitopes by vaccinia virus-expressed gB and gB-N. (a) BHK-21 cells were left uninfected (UI) or infected (1 p.f.u. per cell, 14 h) with either MuHV-4 or a vaccinia virus expressing a GPI-linked gB extracellular domain (VAC-gB). Cell surface gB expression was analysed by flow cytometric staining of pre-fusion gB with mAb BN-1A7, and post-fusion gB with MG-1A12. (b) C57BL/6 mice were infected i.p. with either MuHV-4 or VAC-gB. Sera collected 2 weeks later were used to stain CHO cells either untransfected or transfected with gp70 or GPI-linked gB. Each point shows the median fluorescence intensity for a 1/500 serum dilution from one mouse. The bars show mean values. CHO-gB staining was significantly greater after VAC-gB infection than after MuHV-4 infection (*P*<0.01 by Student's two-tailed *t*-test). In this and subsequent experiments, consistent results were obtained with least two further serum dilutions. (c) Mice were immunized i.p. with either VAC-gB or a control, and 1 month later infected i.n. with MuHV-4. Infectious MuHV-4 in lungs was plaque assayed at 5 days post-challenge. Each point shows the titre of one mouse. The bars show geometric means. (d) Sera from mice infected with either VAC-gB or MuHV-4 were used to neutralize eGFP^+^ MuHV-4 for BHK-21 cell and NMuMG cell infections. Infection was assayed by flow cytometry for eGFP expression 18 h after exposure to virus/antibody. Each point shows mean±sd of triplicate infections. The dashed lines show infection with virus alone.

The MuHV-4 gB can be expressed at cell surfaces independent of other virion components by replacing its transmembrane and cytoplasmic domains with a glycosyl-phosphatidyl-inositol (GPI) anchor (Lopes *et al.*, 2004). VAC-gB-GPI displayed both pre-fusion and post-fusion epitopes, but like transfected gB-GPI (Gillet *et al.*, 2008b) showed a much greater representation of post-fusion epitopes than MuHV-4-infected cells (Fig. 1a). Immunization intraperitoneally (i.p.) with VAC-gB (Fig. 1b) induced a stronger gB-specific serum antibody response than i.p. infection with MuHV-4, as measured by flow cytometric staining of gB-GPI-transfected CHO cells. Immunization also afforded significant protection against intranasally (i.n.) MuHV-4 challenge (Fig. 1c). However, sera from the immunized mice showed no MuHV-4 neutralization (Fig. 1d). Similar results were obtained whether sera were used fresh or after heating (56 °C, 30 min) to inactivate complement, and whether or not reconstituted mouse complement (Sigma Chemical Co.) was added to the antibody-coated virions.

### Expression of gB-N from vaccinia virus

**Fig. 2.**
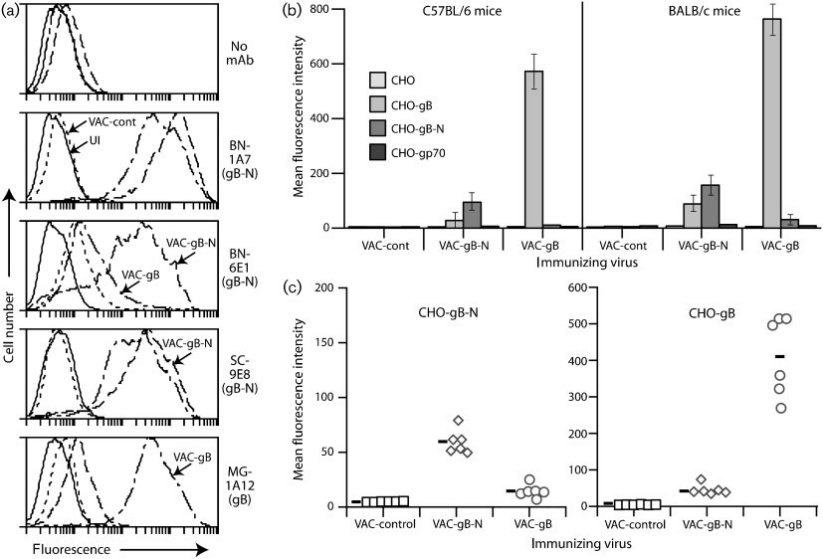
Primary antibody responses to gB vaccines in C57BL/6 and BALB/c mice. (a) BHK-21 cells were left uninfected (UI) or infected (1 p.f.u. per cell, 14 h) with vaccinia virus expressing either the full-length gB extracellular domain (VAC-gB), just its N-terminal domains (VAC-gB-N), or a control insert (VAC-cont). gB expression was analysed by flow cytometry. mAbs BN-6E1 and SC-9E8 recognize neutralizing epitopes on pre-fusion gB. MG-1A12 recognizes a post-fusion epitope that is not contained within gB-N. (b) Sera from mice infected i.p. 1 month previously with VAC-gB, VAC-gB-N or a control virus were pooled from six mice per group and assayed for reactivity to gB, gB-N and gp70 (as a negative control) by flow cytometry of transfected CHO cells. Bars show mean±sd values. For both C57BL/6 and BALB/c mice, VAC-gB-N primed CHO-gB-N-specific responses significantly better than did VAC-gB, and VAC-gB primed CHO-gB-N-specific responses significantly better than did VAC-gB-N (*P*<0.0001 by Student's two-tailed *t*-test). (c) Individual sera from the C57BL/6 mice in (b) were assayed for reactivity to gB and gB-N. Each point shows staining of the relevant CHO cells by serum from one mouse. The bars show mean values. VAC-gB-primed mice showed significantly stronger staining of both CHO-gB (*P*<0.0001) and CHO-gB-N (*P*<0.01), as did VAC-gB-N-primed mice (*P*<0.0001 by Student's two-tailed *t*-test). Each group also showed significantly stronger staining of the cognate form of gB (*P*<0.0001).

We reasoned that gB-N (domains I+II) might more effectively present pre-fusion epitopes than the full-length extracellular domain, as it cannot switch to a post-fusion form. The neutralization epitope recognized by mAb SC-9E8 was presented at least as well by VAC-gB-N as by VAC-gB, as was the distinct neutralization epitope recognized by IgM mAb BN-6E1 (Fig. 2a). Both pooled (Fig. 2b) and individual sera (Fig. 2c) from mice immunized with VAC-gB or VAC-gB-N showed gB/gB-N cross-reactivity, but substantially stronger recognition of the cognate form.

### Protection against primary MuHV-4 lytic replication by VAC-gB-N

**Fig. 3.**
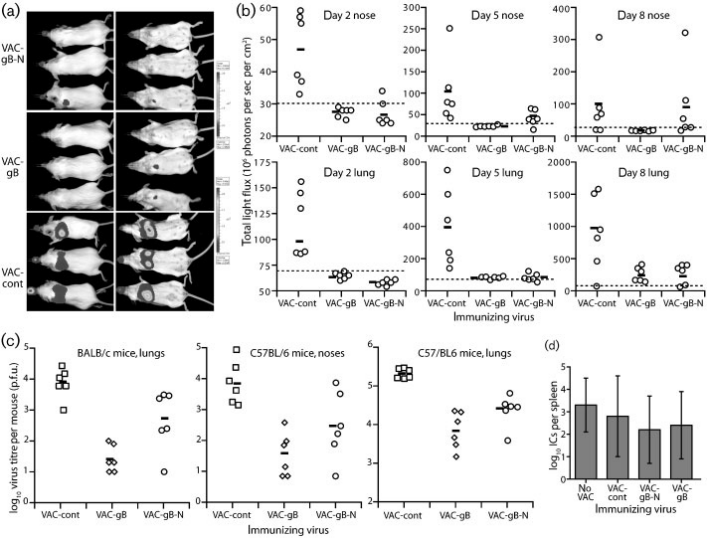
Protection against MuHV-4 lytic replication by vaccination with gB. (a) BALB/c mice were immunized i.p. with vaccinia viruses expressing gB, gB-N or an irrelevant insert (VAC-cont), and 1 month later challenged i.n. (10^4^ p.f.u.) with luciferase^+^ MuHV-4. Viral replication in lungs and noses was monitored by luciferin injection and CCD camera scanning. A representative image is shown of mice 5 days post-challenge. (b) Mice were immunized then infected with luciferase^+^ MuHV-4 as in (a). Each point shows the luciferase signal for one mouse. The bars show mean values. Dashed lines show the lower limits of signal detection. Priming with either VAC-gB or VAC-gB-N significantly reduced luciferase signals in lungs at all time points (*P*<0.03 by Student's two-tailed *t*-test). Luciferase signals in noses were also significantly reduced in VAC-gB-primed mice at days 2 (*P*<0.01) and 5 (*P*<0.04), and in VAC-gB-N-primed mice at day 2 (*P*<0.01). (c) BALB/c or C57BL/6 mice were immunized i.p. with vaccinia virus recombinants then challenged i.n. with MuHV-4 as in (b), but with luciferase^−^ MuHV-4. Lungs and noses were titrated for infectious virus at 6 days post-infection by plaque assay. Each point shows the titre of one mouse. The bars show geometric means. Both BALB/c (*P*<0.04) and C57BL/6 mice (*P*<0.01) lung titres were significantly reduced by priming with either VAC-gB or VAC-gB-N. C57BL/6 mice nose titres were also reduced, although this was only statistically significant for VAC-gB (*P*<0.01). (d) Latent virus loads in spleens were measured by infectious centre assay at 2 months post-challenge of vaccinated C57BL/6 mice. The bars show mean±sd titres of five mice per group.

**Fig. 4.**
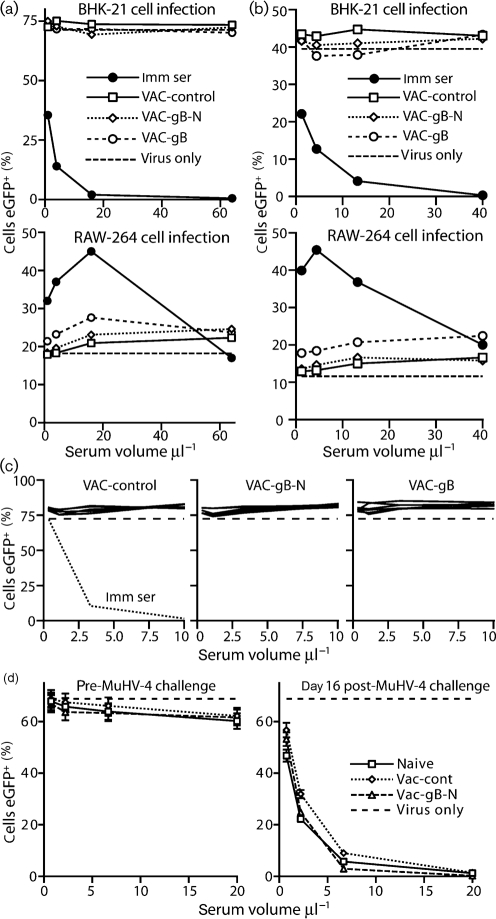
gB-primed sera fail to neutralize MuHV-4. (a) BALB/c mice were primed with vaccinia virus expressing gB, gB-N or a control insert. After 1 month, sera pooled from six mice per group were tested for their capacity to reduce BHK-21 or RAW-264 cell infections by eGFP^+^ MuHV-4, as assayed by flow cytometry 16 h after exposure to virus. Neutralization by sera pooled from MuHV-4-infected mice (Imm ser) is also shown. Dashed lines show the level of infection by virus alone. (b) In a similar protocol to (a), VAC-gB-primed and VAC-gB-N-primed sera, each pooled from six C57BL/6 mice, showed no significant MuHV-4 neutralization. (c) Sera from the mice in (b) were tested individually for neutralization of BHK-21 cell infection. Again none was observed. The dashed line shows neutralization by pooled, MuHV-4-immune sera. (d) Sera pooled from unprimed or VAC-primed mice (*n*=7) were collected either before or 16 days after MuHV-4 challenge and used to neutralize MuHV-4 virions for BHK-21 cell infection. Each line shows mean±sem results. No significant difference was observed between VAC-gB-N-primed and control mice.

Priming with either VAC-gB or VAC-gB-N reduced MuHV-4 lytic replication after i.n. challenge (Fig. 3). Viral luciferase expression (Fig. 3a, b) and plaque assays (Fig. 3c) yielded similar results. VAC-gB protected marginally better than VAC-gB-N, particularly in noses, but both significantly reduced infection compared with controls. As in previous studies (Stevenson *et al.*, 1999b), reducing lytic replication did not affect long-term latency titres: by 2 months post-infection, splenic infectious centre titres were equivalent between vaccinated and unvaccinated mice (Fig. 3d). We were more concerned whether the protection reflected virus neutralization. Despite good gB-specific antibody responses, sera from gB-N-primed mice failed to neutralize MuHV-4 either as pools (Fig. 4a, b) or as individual sera (Fig. 4c). gB-primed sera even showed a modest enhancement of RAW-264 cell infection compared with naive controls (Fig. 4b). Post-challenge serum neutralization titres were also similar between vaccinated and unvaccinated mice (Fig. 4d). Therefore, although both gB and gB-N presented neutralizing epitopes, protection was independent of detectable neutralization.

### Protection by vaccination with gB-N depends on IgG Fc receptors

**Fig. 5.**
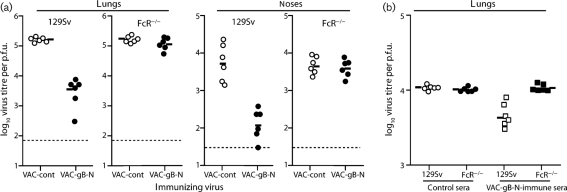
Protection of naive mice and induction of MuHV-4 neutralization by vaccination with a gHL fusion protein. (a) 129Sv×C57BL/6 FcR*γ*^−/−^Fc*γ*RII^−/−^ mice (FcR^−/−^) or 129Sv controls were primed i.p. with vaccinia virus recombinants (10^5^ p.f.u.) and 1 month later challenged i.n. with MuHV-4 (10^4^ p.f.u.). Infectious virus titres in lungs and noses were measured by plaque assay 6 days later. Each point shows the result for one mouse. The bars show geometric means. VAC-gB significantly reduced virus replication in lungs (*P*<0.00001) and noses (*P*<0.04) of 129SV but not FcR^−/−^ mice. (b) Sera were pooled from mice (*n*=10) 3 months after i.p. infection with VAC-gB-N or VAC-cont, then given i.p. to 129Sv or FcR^−/−^ mice (500 μl per mouse) at the same time as i.n. MuHV-4 (5000 p.f.u.). Lungs were titrated for infectious virus by plaque assay 5 days later. Each point shows the result for one mouse, and the bars show geometric means. gB-N-immune sera significantly reduced virus titres in 129Sv mice (*P*<0.0001 by Student's two-tailed *t*-test) but not in FcR^−/−^ mice (*P*=0.86).

Both neutralizing and non-neutralizing passive antibodies reduce MuHV-4 lytic replication by IgG Fc receptor-dependent mechanisms (Wright *et al.*, 2009). The protection by VAC-gB-N appeared to be similar, as it was lost in IgG Fc receptor-deficient mice (Fig. 5a). Immune sera transferred from VAC-gB-N-immunized to naive mice also gave IgG Fc receptor-dependent protection (Fig. 5b).

### Boosting neutralizing antibody responses with VAC-gB and VAC-gB-N

**Fig. 6.**
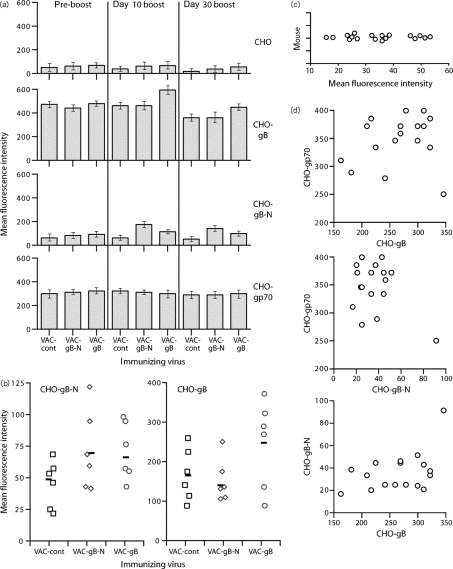
Boosting gB-specific antibody responses by post-exposure vaccination of MuHV-4 carrier mice. (a) C57BL/6 mice were infected i.n. with MuHV-4 and 3 months later boosted i.p. with vaccinia virus expressing either the full-length gB extracellular domain (VAC-gB), just its N-terminal half (VAC-gB-N) or a control insert (VAC-cont). Sera were taken before vaccinia infection (pre-boost), 10 and 30 days later, and tested for gB reactivity by flow cytometric staining of CHO cells expressing gB or gB-N. CHO cells either untransfected or transfected with the MuHV-4 gp70 provided staining controls. Bars show mean±sd fluorescence intensities for sera pooled from six mice each. VAC-gB-N and VAC-gB both significantly increased CHO-gB-N staining at days 10 and 30 compared with VAC-cont (*P*<0.0001 by Student's two-tailed *t*-test). The increase with VAC-gB-N was significantly greater than with VAC-gB (*P*<0.0001). VAC-gB but not VAC-gB-N significantly increased CHO-gB staining at days 10 and 30 compared with VAC-cont (*P*<0.0001). (b) Sera from the individual day 30 boosted mice in (a) were assayed for CHO-gB and CHO-gB-N reactivity. Although boosting increased staining, this did not reach statistical significance (*P*>0.05) because of wide individual variation. Each point shows an individual mouse and the bars show geometric means. (c) C57BL/6 MuHV-4 carrier mice (3 months post-infection) were analysed by flow cytometry for serum binding to CHO-gB-N cells. Each point shows the result for one mouse. (d) A separate set of MuHV-4-infected C57BL/6 mice were compared for serum reactivity to CHO-gB, CHO-gB-N and CHO-gp70 cells. Each point shows the result for one mouse. There was no obvious correlation for individual mice between the responses to each target.

We next tested whether VAC-gB-N could boost neutralizing antibodies in MuHV-4 carrier mice. Previously (Gillet *et al.*, 2007b), it was found that VAC-gB was effective in only a minority of mice. C57BL/6 mice infected i.n. with MuHV-4 were 3 months later boosted i.p. with VAC-gB, VAC-gB-N or an irrelevant VAC control. The mice were bled before boosting, 10 days after (acute response) and 30 days after (longer-term response) (Fig. 6). VAC-gB-N boosted gB-N-specific antibodies significantly better than VAC-gB without improving the recognition of CHO-gB cells, again emphasizing that gB-N and gB are antigenically quite different (Fig. 6a). The magnitude of gB- and gB-N-specific antibody responses varied considerably between individual mice (Fig. 6b). The gB-N-specific antibody responses of non-boosted carriers (Fig. 6c) were also highly variable, and showed no correlation with responses to full-length gB or the MuHV-4 gp70 (Fig. 6d). Thus, even homozygous sibling mice varied considerably in antibody response.

**Fig. 7.**
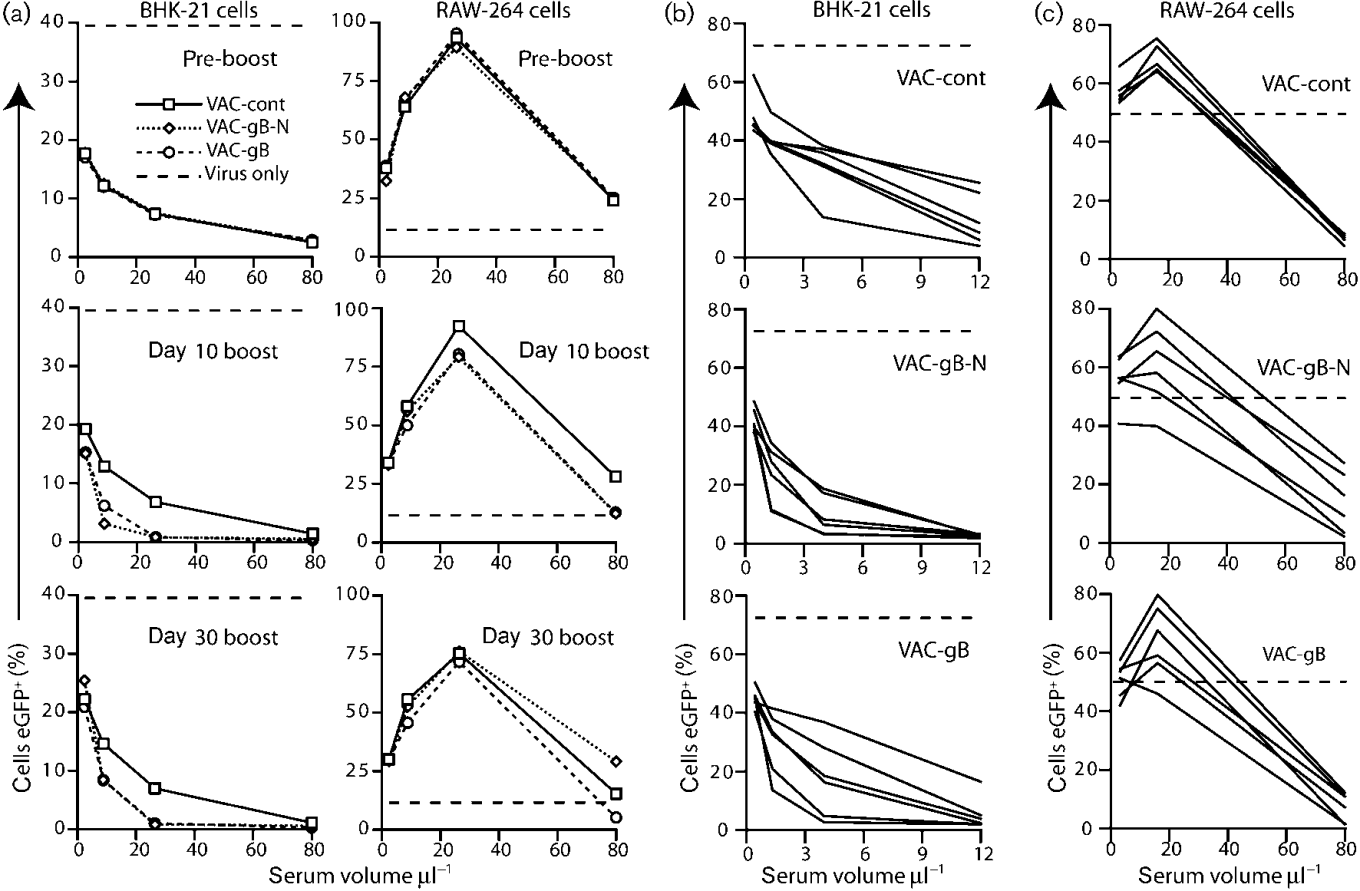
Boosting gB-specific neutralizing antibody responses by post-exposure vaccination of MuHV-4 carrier mice. (a) Sera taken pre-boosting, 10 or 30 days post-boosting with vaccinia viruses expressing the full-length gB extracellular domain (VAC-gB), its N-terminal half (VAC-gB-N) or an irrelevant protein (VAC-cont), were pooled from groups of six C57BL/6 mice each. eGFP^+^ MuHV-4 virions were incubated with dilutions of the pooled sera and then used to infect either BHK-21 cells (0.3 p.f.u. per cell) or RAW-264 cells (3 p.f.u. per cell). Infected cells were enumerated 16 h later by flow cytometry. Dashed lines show infection with untreated virus. Each point gives the result for 10 000 cells. Thus by *χ*^2^ test, the visible reductions in infection with VAC-gB or VAC-gB boosting were all highly significant (*P*<0.0001). The day 30 RAW-264 cell infection differences were not considered significant as they were not maintained over more than 1 serum dilution. (b) Individual sera taken at day 30 post-boosting were analysed for neutralization of BHK-21 cell infection as in (a). With 4 μl serum, both the VAC-gB (*P*<0.05) and VAC-gB-N boosted groups showed a significant reduction in infection (*P*<0.03 by Student's two-tailed *t*-test) compared with the control group. With 1.3 μl serum, only the VAC-gB-N boosted group showed a reduction (*P*<0.03). (c) Sera taken at day 30 post-boosting were analysed for neutralization of RAW-264 cell infection (3 p.f.u. per cell). The boosted groups showed a wider distribution than the unboosted, but no significant reduction.

Pooled C57BL/6 mouse sera (Fig. 7a) neutralized MuHV-4 for BHK-21 cell infection better after boosting with either VAC-gB or VAC-gB-N compared with the control. Boosting improved the neutralization of RAW-264 cell infection only at the 10 day time point. The effects were not large, and individual neutralization titres (Fig. 7b, c) overlapped between groups, consistent with the varied boosting efficacy observed by flow cytometry (Fig. 6). Thus, VAC-gB and VAC-gB-N were both capable of boosting neutralizing antibodies, but worked well in few mice, presumably because few mice made primary gB-specific neutralizing responses. VAC-gB and VAC-gB-N similarly increased significantly gB-N- and gB-specific antibody responses in BALB/c carrier mice (Supplementary Fig. S1, available in JGV Online) with only a small improvement in neutralization (Supplementary Fig. S2, available in JGV Online). As the boosting of neutralization was similar between VAC-gB and VAC-gB-N, the limiting factor appeared to be the composition of the primary antibody response to MuHV-4 rather than the level of subsequent gB-N delivery.

## DISCUSSION

We sought to understand why MuHV-4-infected mice make poor neutralizing antibody responses to gB. Specifically, we tested whether recombinant forms of gB containing known neutralization epitopes could stimulate neutralizing responses in either naive or carrier mice. Immunization with either the full-length gB extracellular domain or its N-terminal half reduced subsequent MuHV-4 lytic replication. However, neither construct elicited detectable primary neutralizing responses, even though both were recognized by a potent neutralizing mAb. Some boosting of neutralizing antibodies in carrier mice was possible, but the effect was not large, there was considerable variation between individual mice, and gB-N was no more effective than gB. We conclude that gB-directed MuHV-4 neutralization is fundamentally limited by the difficulty of targeting the few vulnerable epitopes it presents.

How does an essential component of the MuHV-4 entry complex avoid being a good neutralization target? It seems likely that post-endocytic conformation changes allow key gB epitopes to remain hidden on extracellular virions. Thus, while gB-N binds to cells (Gillet & Stevenson, 2007b), this must occur downstream in entry as blocking heparan sulfate binding by gp70 and gH/gL largely abrogates virion binding (Gillet *et al.*, 2009b). The increased susceptibility of gL^−^ virions to gB-directed neutralization (Gillet *et al.*, 2009a) implies that gH/gL helps to hide gB; the regulation of virion binding by gp150 (de Lima *et al.*, 2004) suggests that it could also be involved. While the neutralization epitope defined by mAb SC-9E8 is accessible it is evidently difficult to target, much like broadly neutralizing human immunodeficiency virus epitopes (Burton *et al.*, 2005). Such resistance to neutralization may be a common feature of persistent viruses.

Herpes simplex virus (HSV) and human cytomegalovirus (HCMV) may be more easily neutralized *in vitro* by gB-specific antibodies (Cranage *et al.*, 1986; Highlander *et al.*, 1988; Utz *et al.*, 1989) because they fuse at the plasma membrane rather than in late endosomes: if gB conformation switches are reversible as for vesicular stomatitis virus glycoprotein G (Roche *et al.*, 2007) and some switching precedes actual fusion, then mAbs specific for the post-fusion HSV or HCMV gB could lock it non-productively in that form. Such a mechanism would be consistent with HSV and HCMV neutralization epitopes mapping to prominent features of post-fusion gB (Heldwein *et al.*, 2006). In contrast, endocytic entry hides the MuHV-4 gB conformation changes from antibody. HCMV may be protected partly by competition between neutralizing and non-neutralizing antibodies (Speckner *et al.*, 1999), but viral transmission would also segregate plasma membrane fusion from the antibody in immune donors, thereby protecting gB epitopes revealed after cell binding.

Reducing viral lytic replication may reduce transmission events associated with primary infection, but optimally protecting naive subjects against infection by virus carriers is likely to require mucosal vaccination, either boosting IgA responses in carriers or generating primary IgA responses in naive subjects. First it is necessary to induce good neutralization. Attention so far has focussed on IgG, since IgG responses are more readily elicited by vaccination. Alpha- and betaherpesvirus gB vaccine studies (Willey *et al.*, 1988; Nazerian *et al.*, 1992; Pass *et al.*, 2009) have generally not distinguished neutralization from IgG Fc receptor-dependent effector functions. This first description of gB immunization against a gammaherpesvirus gave protection consistent with the alpha- and betaherpesvirus studies, but showed that neutralization was not involved. Therefore, it seems unlikely that vaccination with gB alone will reduce infection prevalence.

## METHODS

### Mice.

C57BL/6, BALB/c, Sv129 (Harlan UK Ltd) and C57BL/6×Sv129 FcR*γ*^−/−^Fc*γ*RII^−/−^ mice (Taconic Europe) were housed at the Cambridge University Department of Pathology and infected when 6–8 weeks old under the Home Office Project Licence 80/1992. All experiments conformed to local and national ethical regulations. MuHV-4 (10^4^ p.f.u.) was given i.n. under general anaesthesia. Vaccinia viruses (10^6^ p.f.u.) were given i.p. For luciferase imaging, mice were injected i.p. with luciferin (2 mg per mouse), anaesthetized with isoflurane and scanned with an IVIS Lumina (Caliper Life Sciences). Quantitative comparisons were made by using Living Image software. Sera were collected from tail veins, or by cardiac puncture under terminal anaesthesia.

### Cells.

BHK-21 fibroblasts (ATCC CCL-10), TK^−^143 cells (ATCC CRL-8303), NMuMG epithelial cells (ATCC CRL-1636), NIH-3T3-CRE cells (Stevenson *et al.*, 2002), RAW-264 monocytes (ATCC TIB-71), CHO-K1 cells (ATCC CCL-61) and CHO-K1 derivatives expressing either gp70, the main product of ORF4 (Gillet *et al.*, 2007a), a fusion protein of the gH and gL extracellular domains with a GPI anchor (Gillet *et al.*, 2007c), or a GPI-linked gB extracellular domain (CHO-gB) (Lopes *et al.*, 2004), were grown in Dulbecco's modified Eagle's medium (DMEM; Invitrogen Corporation) supplemented with 10 % FCS (PAA Laboratories), 2 mM glutamine, 100 U penicillin ml^−1^ and 100 μg streptomycin ml^−1^. To make CHO-gB-N cells, the coding sequence for gB N-terminal to its furin cleavage site (aa 1–423) was cloned upstream of a GPI anchor attachment site as for full-length gB (Lopes *et al.*, 2004). CHO-K1 cells transfected with the gB-N expression plasmid were selected with hygromycin (250 μg ml^−1^).

### Viruses.

All MuHV-4 derivatives were generated from a BAC-cloned genome (Adler *et al.*, 2000). Luciferase expression from an ectopic MuHV-4 lytic promoter has been described previously (Milho *et al.*, 2009). The MuHV-4 BAC contains an HCMV IE1 promoter-driven eGFP expression cassette. However, this is poorly transcribed in some cell types (Rosa *et al.*, 2007). Therefore to assay *in vitro* infection, we inserted into the MuHV-4 genome a separate, intergenic eGFP expression cassette with an EF1*α* promoter. We first mutated the internal *Bgl*II site of the EF1*α* promoter in pBRAD from AGATCT to AGGTCT by overlap PCR, then PCR-amplified the modified promoter, adding *Bgl*II and *Eco*RI sites to its respective 5′ and 3′ ends, and cloned upstream of a poly(A) site in pSP73 (May *et al.*, 2005). The eGFP coding sequence was PCR-amplified from pEGFP-N3 (Clontech), adding 5′ *Eco*RI and 3′ *Xho*I sites, and cloned into *Eco*RI/*Sal*I sites between the EF1*α* promoter and poly(A) site. The resulting eGFP expression cassette was then subcloned as a blunted *Bgl*II–*Xho*I fragment into the *Mfe*I site of a 3.4 kb *Bgl*II genomic clone, between the 3′ ends of ORFs 57 and 58 (May *et al.*, 2005). Inserts here have no obvious effect on MuHV-4 replication (Bennett *et al.*, 2005). The eGFP expression cassette and its genomic flanks were then subcloned into the pST76K-SR shuttle vector and recombined into the MuHV-4 BAC by standard methods (Adler *et al.*, 2000). Recombinant clones were identified and checked for genome integrity by restriction enzyme mapping. Infectious virus was recovered by transfecting BAC DNA into BHK-21 cells. The BAC cassette was then removed by passage through NIH-3T3-CRE cells. Virus stocks were grown in BHK-21 cells. Virions were harvested from infected cell supernatants by ultracentrifugation, and cell debris removed by low speed centrifugation (de Lima *et al.*, 2004).

Vaccinia viruses expressing a GPI-linked gB extracellular domain or gH/gL fusion protein have been described previously (Gillet *et al.*, 2007b). To make VAC-gB-N, the coding sequence for gB aa 81–423 was amplified by PCR, digested with *Xba*I at a site internal to gB and cloned as a blunt *Xba*I fragment into the *Sna*BI/*Xba*I sites of gB in pBRAD-gB. The predicted gB signal sequence is aa 1–24, and *Sna*BI cuts after residue 22, so the coding sequence for residues 23–25 was included in the 5′ PCR primer, thereby reconstituting the native signal sequence without residues 26–80. The modified gB-N coding sequence plus C-terminal GPI attachment site was PCR-amplified from pBRAD-gB-N using 5′ *Nhe*I-restricted and 3′ *Hin*dIII-restricted primers and cloned into the *Nhe*I/*Hin*dIII sites of the vaccinia recombination plasmid pMJ601 (Davison & Moss, 1990). pMJ-601-gB-N-GPI was then transfected into vaccinia virus-infected TK^−^143 cells. Thymidine kinase-deficient recombinants were selected by passage in 25 μg 5′-bromo-2′-deoxyuridine (Sigma Chemical Co.) ml^−1^, identified by *β*-galactosidase expression using X-Gal, and plaque purified. Virus stocks were grown and titrated using TK^−^143 cells. As a TK^−^ control, we used vaccinia virus expressing the mouse invariant chain coding sequence with ovalbumin residues 323–339 substituted for the CLIP peptide (Smith *et al.*, 2006).

### Virus titres.

Virus stocks and *ex vivo* samples were titrated for infectivity by plaque assay on BHK-21 cells (de Lima *et al.*, 2004). Lungs and noses were removed from mice post-mortem, freeze–thawed, then homogenized in DMEM. Nose samples included the turbinates and nasal septum, which contain all the nasal luciferase signal of mice infected with luciferase^+^ MuHV-4 (Milho *et al.*, 2009). Serial dilutions of each sample were incubated (2 h, 37 °C) with BHK-21 cell monolayers, then overlaid with DMEM plus 0.3 % carboxymethylcellulose. After 4 days the monolayers were fixed in 4 % formaldehyde, stained with 0.1 % toluidine blue and plaques were counted with a plate microscope.

### Neutralization assays.

EF1*α*-eGFP MuHV-4 was incubated with serum dilutions (2 h, 37 °C), then added to BHK-21 fibroblasts or RAW-264 monocytes. After 2 h, phosphonoacetic acid was added (100 μg ml^−1^) to prevent lytic spread. After 16 h, the cells were harvested and assayed for eGFP expression by flow cytometry. In preliminary experiments, virus titres by EF1*α*-driven eGFP expression in BHK-21 cells equalled or exceeded plaque assay titres, and in RAW-264 cells equalled or exceeded BAC cassette-associated eGFP expression maximized by lipopolysaccharide treatment (Rosa *et al.*, 2007).

### Flow cytometry.

Cells exposed to eGFP^+^ viruses were washed twice in PBS and analysed directly for green channel fluorescence. For specific staining, cells were incubated (1 h, 4 °C) with MuHV-4 gB-specific mAbs or with immune sera, followed by fluorescein-conjugated rabbit anti-mouse IgG pAb (Dako Cytomation). mAbs BN-1A7 (IgG2a), BN-6E1 (IgM) and SC-9E8 (IgG2a) all recognize epitopes in gB-N and are specific for pre-fusion gB, whereas MG-1A12 is specific for post-fusion gB (Gillet *et al.*, 2008c). All cells were washed twice in PBS after each antibody incubation and analysed on a FACS Scan that runs the CellQuest software (BD Biosciences).

## Supplementary Material

[Supplementary Figures]
