# Supplementary material for: Vaccination with murid herpesvirus-4 glycoprotein B reduces viral lytic replication but does not induce detectable virion neutralization
Source: J Gen Virol. 2010 Oct;91(Pt 10):2542–52. doi: 10.1099/vir.0.023085-0 (PMC3052599; doi:10.1099/vir.0.023085-0)
Supplement: [Supplementary Figures] [file supp_91_10_2542__index.html]

 Vaccination with murid herpesvirus-4 glycoprotein B reduces viral lytic replication but does not induce detectable virion neutralization -- May and Stevenson 91 (10): 2542 Data Supplement - Supplementary Figures -- Journal of General Virology

## 

### Vaccination with murid herpesvirus-4 glycoprotein B reduces viral lytic replication but does not induce detectable virion neutralization, by J. S. May and P. G. Stevenson

*Journal of General Virology* vol. **91**, part 10, pp. 2542 - 2552

**Supplementary Fig. S1.** Boosting gB-specific antibody responses by post-exposure vaccination of BALB/c MuHV-4 carrier mice

**Supplementary Fig. S2.** Boosting gB-specific neutralizing antibody responses in BALB/c carrier mice   
  
 [Single PDF file]  (599 KB)

  
  
