# Supplementary material for: Vaccination with murid herpesvirus-4 glycoprotein B reduces viral lytic replication but does not induce detectable virion neutralization
Source: J Gen Virol. 2010 Oct;91(Pt 10):2542–52. doi: 10.1099/vir.0.023085-0 (PMC3052599; doi:10.1099/vir.0.023085-0)
Supplement: [Supplementary Figures] [file supp_91_10_2542__1.pdf]

Supplementary Figure 1

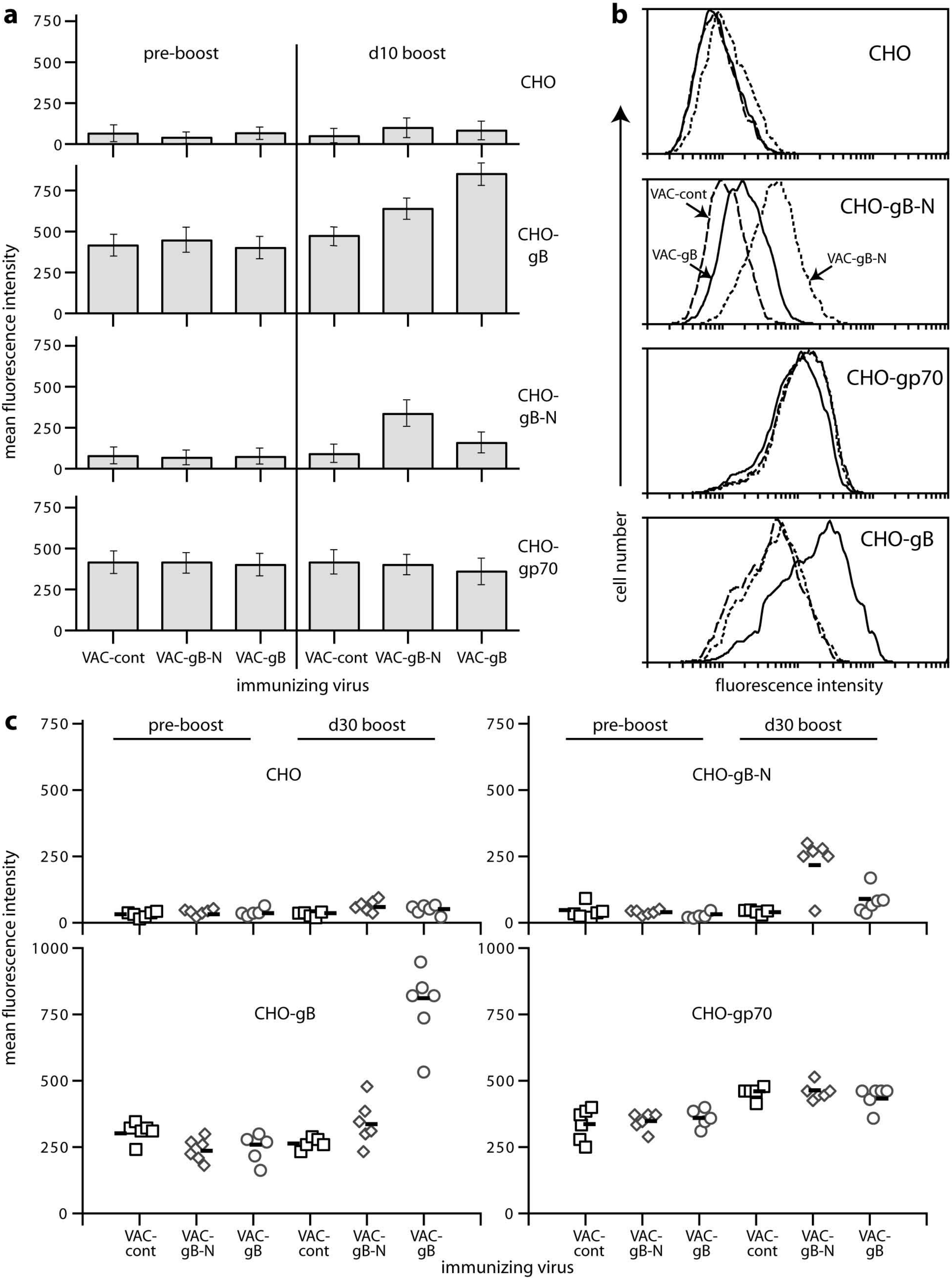

Supplementary Figure 2

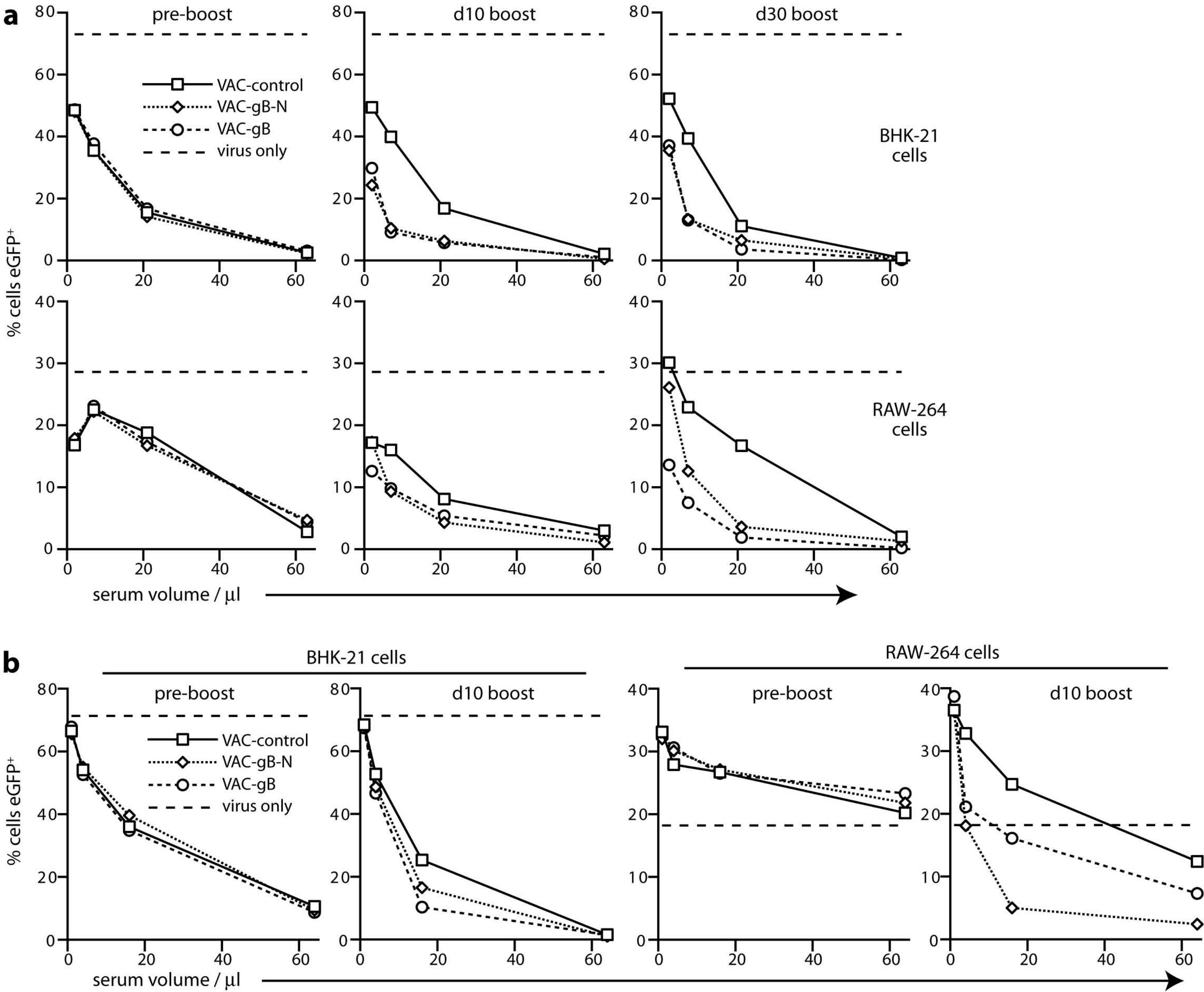

Supplementary Fig. S1. Boosting gB-specific antibody responses by post-exposure vaccination of BALB/c MuHV-4 carrier mice. (a) BALB/c mice were infected i.n. with MuHV-4 and 3 months later infected i.p. with vaccinia viruses expressing either the gB extracellular domain (VAC-gB), its N-terminal half (VAC-gB-N) or an irrelevant insert (VAC-cont). Sera taken before vaccinia infection or 10 days afterwards were tested for gB reactivity by flow cytometric staining of CHO cells expressing gB or gB-N. Untransfected and gp70-transfected CHO cells provided controls. The bars show mean±SD fluorescence intensities for sera pooled from six mice each. VAC-gB-N and VAC-gB both significantly increased CHO-gB-N staining compared with VAC-cont ($P<0.0001$ by Student's two-tailed *t*-test). The increase with VAC-gB-N was significantly greater than with VAC-gB ($P<0.0001$). Both VAC-gB and VAC-gB-N also significantly increased CHO-gB staining at day 10 compared with VAC-cont, with VAC-gB giving significantly greater boosting than VAC-gB-N ($P<0.0001$). (b) Flow cytometric staining profiles are shown for pooled sera ($n=6$) taken at day 30 after VAC-gB, VAC-gB-N or VAC-cont boosting of BALB/c mice. (c) Sera from individual mice taken at day 30 after vaccinia virus boosting were assayed for reactivity to CHO, CHO-gB, CHO-gB-N and CHO-gp70 cells by flow cytometry. Each point shows the mean fluorescence intensity for one mouse. The bars show mean values. VAC-gB significantly boosted CHO-gB-specific ($P<0.001$) but not CHO-gB-N-specific responses ($P=0.05$). VAC-gB-N significantly boosted both CHO-gB-specific ($P<0.02$) and CHO-gB-N-specific responses ($P<0.001$). gB boosting had no significant effect on CHO or CHO-gp70 staining.

Supplementary Fig. S2. Boosting gB-specific neutralizing antibody responses in BALB/c carrier mice. (a) Pooled sera from MuHV-4 carrier mice ($n=6$) boosted or not with VAC-gB, VAC-gB-N or a control virus (VAC-cont) were compared for reductions of BHK-21 (1 p.f.u. per cell) and RAW-264 (2 p.f.u. per cell) infections by eGFP+ MuHV-4. $eGFP^+$ cells were enumerated by flow cytometry. The dashed lines show infections without serum (virus only). Each point gives the result for 10000 cells, so the reductions achieved with day 10 and day 30 VAC-gB and VAC-gB-N boosted sera were all highly significant by χ^2 test ($P<0.0001$). (b) Boosting the carrier mice with VAC-gB or VAC-gB-N gave small but significant ($P<0.0001$) improvements in the neutralization of BHK-21 (1 p.f.u. per cell) and RAW-264 cell infections (3 p.f.u. per cell) by pooled sera.

May, J. S. and Stevenson, P. G. (2010). Vaccination with murid herpesvirus-4 glycoprotein B reduces viral lytic replication but does not induce detectable virion neutralization. *J Gen Virol* **91**, 2542–2552.
